# Visually Impaired OLder people's Exercise programme for falls prevenTion (VIOLET): a feasibility study protocol

**DOI:** 10.1136/bmjopen-2016-011996

**Published:** 2016-08-02

**Authors:** Dawn A Skelton, Cathy Bailey, Denise Howel, Mima Cattan, Vincent Deary, Dot Coe, Lex D de Jong, Sheena Gawler, Joanne Gray, Rosy Lampitt, Jennifer Wilkinson, Nicola Adams

**Affiliations:** 1 Institute of Allied Health Research, School of Health and Life Sciences, Glasgow Caledonian University, Glasgow, UK; 2 Faculty of Health and Life Sciences, Department of Public Health and Well Being, International Ageing, Newcastle upon Tyne, UK; 3 Institute of Health and Society, Newcastle University, Newcastle upon Tyne, UK; 4 Department of Public Health and Well Being, Faculty of Health and Life Sciences, Northumbria University, Newcastle upon Tyne, UK; 5 Department of Psychology, Northumbria University, Newcastle upon Tyne, UK; 6 Department of Sport, Exercise and Rehabilitation, Northumbria University, Newcastle upon Tyne, UK; 7 Institute of Allied Health Research, School of Health and Life Sciences, Glasgow Caledonian University, Glasgow, UK; 8 Department of Healthcare, Northumbria University, Newcastle upon Tyne, UK; 9 Newcastle Clinical Trials Unit, Newcastle University Newcastle upon Tyne, Newcastle upon Tyne, UK

**Keywords:** Accidential Falls, Visually impaired older person, Secondary (or tertiary) prevention or accident prevention, Exercise therapies, Feasibility studies

## Abstract

**Introduction:**

In the UK, 1 in 5 people aged 75 and over live with sight loss. Visually impaired older people (VIOP) have an above average incidence of falls and 1.3–1.9 times more likely to experience hip fractures, than the general population. Older people with eye diseases are ∼3 times more likely than those with good vision, to limit activities due to fear of falling. This feasibility study aims to adapt the group-based Falls Management Exercise (FaME) programme to the needs of VIOP and carry out an external pilot trial to inform the design of a future definitive randomised controlled trial.

**Methods and design:**

A UK based 2-centre mixed methods, randomised, feasibility study will be conducted over 28 months. Stakeholder panels, including VIOP, will make recommendations for adaptations to an existing exercise programme (FaME), to meet the needs of VIOP, promoting uptake and adherence, while retaining required effective components of the exercise programme. 80 VIOP aged 60 and over, living at home, ambulant with or without a walking aid, will be recruited in Newcastle (n=40) and Glasgow (n=40) through National Health Service (NHS) Trusts and third sector partners. Participants randomised into the intervention arm will receive the adapted FaME programme. Participants randomised into the control arm will continue with usual activity. Outcomes are, recruitment rate, adherence and validated measures including fear of falling and quality of life. Postintervention in-depth qualitative interviews will be conducted with a purposive sample of VIOP (N=10). Postural stability instructors will be interviewed, before trial-specific training and following the intervention.

**Ethics and dissemination:**

Ethics approval was secured through the National Research Ethics Service (NRES) Committee North East, Newcastle and North Tyneside 2. Glasgow Caledonian University was approved as a non-NHS site with local ethics approval. Findings will be disseminated through peer-reviewed journals, national and international conferences.

**Trial registration number:**

ISRCTN16949845.

**Strengths and limitations of this study**

- Addresses falls prevention intervention in an often excluded population, older people with visual impairment, with a known efficacious falls prevention intervention (Falls Management Exercise—FaME).
- Working with older people with visual impairment to adapt FaME to their needs, a programme so far only tested on sighted people.
- As a feasibility study run in two study sites, it does not have the power to show effectiveness but will allow informed planning for a definitive randomised controlled trial in the future if shown to be feasible.

## Background

One in eight people in the UK aged over 75 and one in three aged over 90, live with significant sight loss.^1^ Visually impaired older people (VIOP) are more likely to move into residential settings than sighted peers, be physically dependent and have poorer quality of life.^2–4^ Vision impairment (VI) is associated with a loss of function in activities of daily living.^5^ ^6^ A UK report by Visibility^7^ found that older people are highly likely to avoid activity because of their VI. Anxiety and depression are also common in those with VI and this also leads to reduced activity.^8^

Falls are seldom due to a single cause.^9^ ^10^ Poor vision has been identified as being particularly related to the recurrence of falls^11^ although VI is not independently associated with higher incidence of falls.^12^ Visual acuity, contrast sensitivity and visual field defects have been identified as important features of poor vision and VI related to falls risk.^13^ ^14^ VIOP have a 1.7 times higher risk of falling than the general population and report more contact with their general practitioner (GP) and more hospital and nursing home admissions than their peers who do not have VI.^14^ ^15^ Estimates of the number of falls attributed to VI suggest that 8% of falls-related hospital admissions are likely to occur in visually impaired people, accounting for 21% of the total cost of treating accidental falls.^15^ These estimates may hide wide geographical variation and falls attributed to VI are underestimated, as reasons for falling may not be known or recorded and costs to GPs may not be included in treatment costs.^15^

Falls in older people cost the National Health Service (NHS) ∼4.6 million per day.^16^ Evidence suggests multifactorial falls intervention programmes are effective in reducing falls among older people and include tackling underlying health problems, initiating strength and balance training, offering home modifications and checking footwear.^17^ ^18^ One study has considered both occupational therapy and physiotherapy intervention in people with visual impairment and saw a reduction in falls.^19^ In the UK, the National Service Framework for Older People^20^ suggested that falls should be addressed in NHS. In 2004, the National Institute for Health and Care Excellence (NICE) guidelines on prevention and assessment of falls in older people^21^ suggested that by 2005, all NHS boards in the UK should provide a falls service. A survey in 2008^22^ highlighted that just half of participating falls clinics in the UK, assessed for VI but there was little information about how to modify falls prevention programmes for VIOP.

Fear of falling (FoF), with or without a fall, is also common in VIOP and can lead to a cycle of restricting daily activity and mobility with loss of confidence, diminishing physical and mental assets, reduced social participation and overall quality of life.^23–25^ FoF is a broad term encompassing fear, anxiety, impaired perception of ability to walk safely without falling and loss of confidence.^26^ FoF is a significant predictor of a future fall.^27^ It is clear, in therapists and participants, that there is a distinction between proper caution around activity and an overcautious/fear-avoidance cycle that perpetuates disability.^28^ A recent review of FoF^29^ suggests that FoF contributes to falls risk on top of actual gait or balance problems. Exercise can reduce FoF,^30^ at least in the short term, however FoF can also be a barrier to uptake of exercise programmes. Research suggests that perceived risk, stigma, lack of awareness among health professionals, lack of appropriate supporting materials and FoF may be barriers to exercise attendance.^7^ ^23^ Enabling factors could include peer acceptability, appropriate supporting material with demonstration, sensitive explanation, carer involvement and individually tailored interventions.^7^ ^23^

The study draws on the learning from a number of previous studies, including the Falls Management Exercise (FaME)^31^ programme, the visually impaired person (VIP) trial (prevention of falls in people aged ≥75 with severe visual impairment),^19^ and the recently completed VIP2UK pilot study (Application Reference Number: UKCRN ID 10883) adapted from the Otago home-based programme to increase adherence in VIOP.^23^ It is known that adherence to the Otago programme in VIOP was significantly lower than older people without significant visual impairment (only 18% VIOP completed all home exercise sessions over a year period^19^) and this may have been due to lack of confidence exercising at home without supervision. Although the VIP2UK study showed better compliance to exercise with additional support strategies (mentors, extra phone calls, audio exercise clips and embedding exercises into daily living) being in place but still low compared with previous studies of the Otago programme in general older adults.^23^

The FaME group-based programme was chosen as the preferred exercise intervention as a recent Royal College of Physicians report showed that 54% of falls services have trained postural stability instructors (PSIs) delivering the FaME programme in groups.^32^ A 6-month programme of FaME exercises has also been shown to significantly increase habitual physical activity in older people recruited through primary care, by 15 min of moderate to vigorous activity a day even at 12 months postintervention, as well as reducing falls.^33^

This paper describes the protocol for the Visually Impaired OLder People's Exercise programme for falls prevenTion (VIOLET), feasibility study (V.1.1, 2 February 2015). The study was funded by the National Institute for Health Research (NIHR) Public Health Research (PHR) Programme.

The rationale for the study is to provide an opportunity for VIOP to contribute to the adaptation of a group-based falls prevention programme that is prevalent in falls services across the UK. This should facilitate an acceptable, feasible and appealing intervention that will improve uptake and adherence to a known effective intervention.

## Aim of the study

To conduct a mixed-methods feasibility and pilot study to inform the design and conduct of a future definitive multicentre randomised controlled trial (RCT) of an adapted group-based exercise programme to prevent falls and reduce FoF among VIOP.

## Objectives

### Feasibility study

1. Explore VIOP’s ability to act as lay partners in a study to develop a condition-appropriate intervention.
2. Explore VIOP’s reasons for participating/not participating in the exercise programme and for those who participate, their experiences of the feasibility study procedures.
3. Identify the feasibility, delivery and acceptability of candidate outcome measures for the future RCT (validated questionnaires/interview methods to measure FoF, activity avoidance, well-being/quality of life, anxiety, depression, loneliness and the number of falls) and VIOP centred/identified outcome measures.
4. Explore the capacity to deliver the adapted exercise programme.
5. Examine delivery (fidelity) and compliance of the exercise intervention.

### Randomised control pilot

1. Assess recruitment of older people with visual impairment (VIOP) and their willingness to be randomised into the adapted falls prevention exercise programme.
2. Test the methodology and to provide outcome data to inform sample size calculations for a definitive RCT.
3. Assess the feasibility of collecting service-use data for an economic evaluation of the intervention in a future RCT.
4. Develop a manualised intervention protocol and training package for a definitive RCT.

## Study governance

A Trial Management Group (TMG), including the chief investigator, study statistician, trial manager and database manager is responsible for overseeing the progress of this feasibility study. The day-to-day management is coordinated by Dr Rosy Lampitt, Trial Manager, Newcastle Clinical Trials Unit. A Trial Oversight Committee (TOC), with an independent Chair and including external members independent of the study, fulfils the combined role and responsibilities of a Data Monitoring and Ethics Committee (DMEC) and Trial Steering Committee (TSC). The TOC acts as the oversight body for this study on behalf of the sponsor and funder, safeguards the interests of study participants, assesses the safety and efficacy of the intervention, and monitors the overall conduct of the study. The TOC also protects the validity and credibility of the study, and provides advice through its independent Chair to the TMG, sponsor and funder on all aspects of the study.

## Methods

### Study design

This is a two-centre randomised feasibility study of an adapted exercise programme for VIOP with embedded qualitative evaluation. The comparison is between a 12-week exercise programme (1-hour session per week plus two home-based sessions progressing from 30 min to 2 hours) and usual activities. Stakeholder panels of VIOP meeting at the two sites will discuss what adaptations to the FaME programme^31^ are desirable and necessary to make the programme more accessible to VIOP.

### Study setting, recruitment and screening

**Figure 1:**
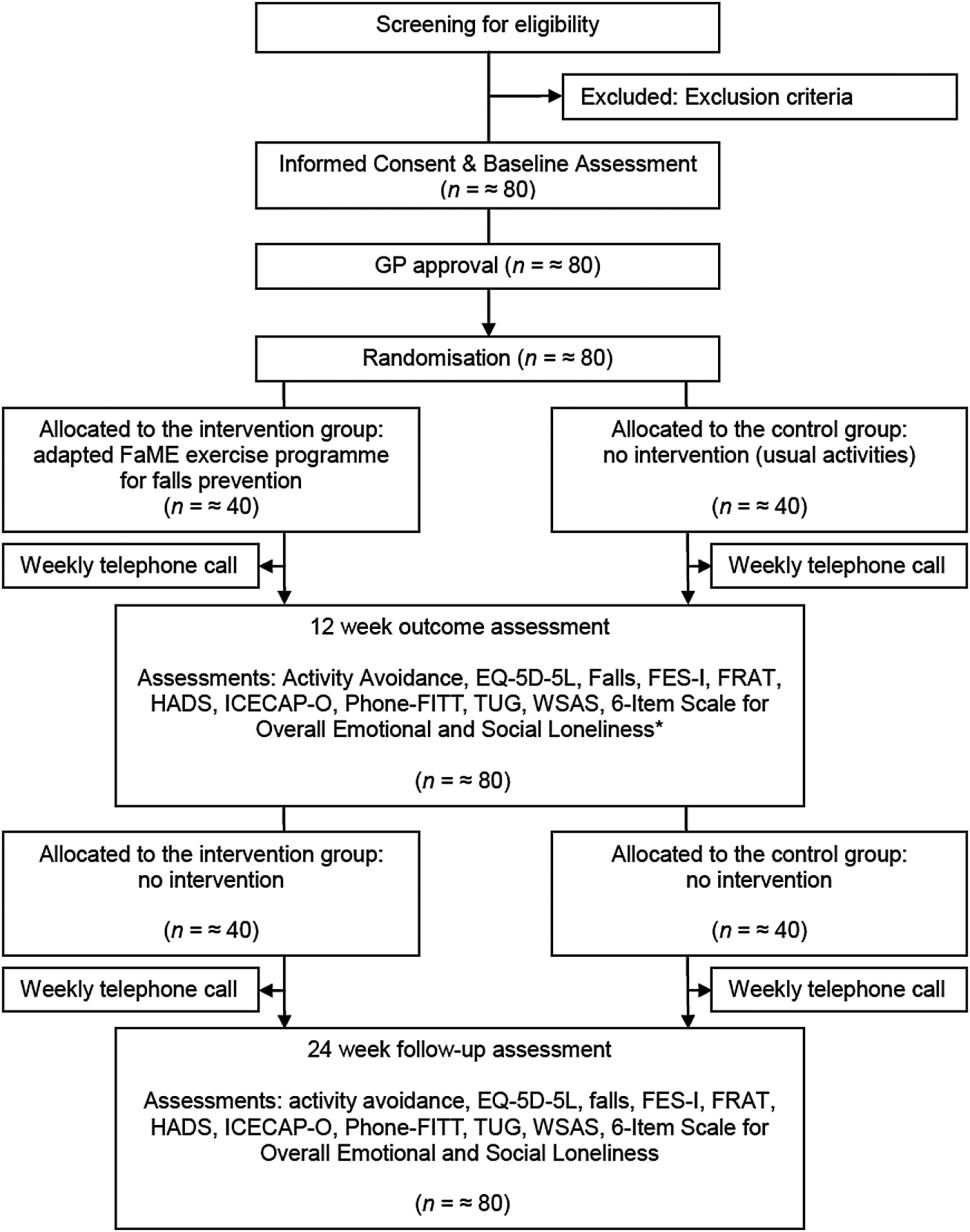
Planned flow of participants throughout the VIOLET study. FaME, Falls Management Exercise; FES-I, Falls Efficacy Scale International; FRAT, Falls Risk Assessment Tool; GP, general practitioner; HADS, Hospital Anxiety and Depression Scale; TUG, Timed Up & Go test; VIOLET, Visually Impaired OLder people's Exercise programme for falls prevenTion; WSAS, Work and Social Adjustment Scale.

Figure 1 provides a consort diagram of planned flow of participants for this study.

Eighty community-living VIOP aged 60 and over will be recruited (40 from each of the two study sites). In Newcastle, participants will be recruited from the Royal Victoria Infirmary's (RVI) Eye Centre and from Newcastle Society for the Blind's (NSBP) membership. In Glasgow, participants will be recruited from the Glasgow Caledonian University Low Vision Clinic and from the vision charity, Visibility Glasgow. Invitation letters and information about the study in large print or audio recorded (in the participant's preferred format) will be sent out through the Low Vision Units and the organisations for visually impaired people or handed out by the low vision specialist/activities coordinators. The participant information will be designed in consultation with the stakeholder panel and checked to ensure that it meets Royal National Institute for the Blind (RNIB) standards in terms of suitability for those with visual impairment.

VIOP who express interest in participating in the study will be screened for eligibility by a researcher over the telephone or on site, based on the inclusion/exclusion criteria below. Once informed consent has been gained, the participant's GP will be approached, via a letter explaining the inclusion and exclusion criteria, to make a judgement regarding the medical fitness of the participant to take part in the exercise intervention. A screening log will be completed by the research staff. It will document the outcome of the screening contact and record any reasons for screen failure. For participants who decline participation, the screening log will document any reasons available for non-participation. The log will also ensure potential participants are only approached once.

### Inclusion criteria

Participants will be aged 60 years and over and attending a low vision clinic and/or are members of organisations for the visually impaired. They will also be living in their own home, able to walk indoors without the help of another person but may use a walking aid, such as a stick, able to walk outdoors but may need the help of another person and/or a walking aid and be physically able to take part in a group exercise class. The definition of visual impairment in the inclusion criteria is deliberately broad and pragmatic, in order to apply findings to the whole spectrum of older participants who may have vision difficulties and be at risk of falls. The extent to which participants perceive their visual impairment function and its impacts on daily living and any changes to their perceived visual function after the interventions will be gathered within interviews and will inform the methodology of the definitive trial.

### Exclusion criteria

Those excluded will: (1) be unable to understand or follow simple movement instructions in English; (2) have acute or uncontrolled medical problems which the participant's GP considers would exclude them from undertaking the exercise programme (eg, uncontrolled heart disease, poorly controlled diabetes, acute systemic illness, neurological problems, severe chronic obstructive pulmonary disease), in addition to visual impairment; (3) have conditions requiring a specialist exercise programme, for example, uncontrolled epilepsy, severe neurological disease or impairment, unable to maintain a seated upright position or unable to move independently indoors; and (4) be currently involved in other falls prevention exercise programmes (but not excluding walking programmes), investigational studies or trials.

### Consent procedures

Signed or recorded verbal consent to participate in the feasibility study (as appropriate for each VIOP participant) will be sought. If the VIOP is unable to sign the consent form, verbal consent will be witnessed by a third party, who will sign the witness section of the consent form. The participant will specifically consent to: (1) their GP being contacted regarding their medical fitness and being informed of their participation in the study; and (2) being informed that they will not be recruited if the GP considers the study to be a contraindication. The right to refuse to participate without giving reasons will be respected. It will also be explained that if it is felt the intervention is or has the potential to cause significant harm to a participant, then for safety reasons, the participant involvement will be discontinued. The information sheet and consent form for the study will be available only in English. It was decided to exclude participants who are unable to understand simple instructions in English, as they would not be able to understand the movement instructions given during the exercise sessions, if randomised to the intervention arm of the study.

### Randomisation

A blocked allocation (permuted random blocks of variable length) system will be used to allocate participants to the two groups (block size will not be disclosed to the investigators) in a 1:1 ratio to intervention and control groups. Randomisation will be stratified by centre. Randomisation will be administered centrally via Newcastle Clinical Trials Unit using a secure web-based system. The principal investigator at site, or individual with delegate authority, will access the web-based system. Participant screening ID, initials and gender will be entered into the web-based system, which will return the allocation status. Participants will be informed by telephone, of their allocated treatment group following randomisation.

### Study intervention

This two-centre randomised feasibility study incorporates a control and an intervention condition.

### Control arm

Those participants who are randomised to the control group will receive no intervention but will be offered an opportunity to take part in an equivalent falls prevention exercise class, provided by local organisations currently delivering these classes after the follow-up period.

### Intervention arm

For the intervention arm, local qualified PSIs working at each site will be trained by the research team members with PSI expertise (DAS and SG) on adapting the exercises for people with visual impairment, and following recommendations from the stakeholder panels. The PSIs will also receive visual impairment training from one of the partner organisations for people with visual impairment (Visibility).

The exercise class (the intervention) will run weekly over 12 weeks, with each session lasting up to 1 hour with an opportunity to socialise after the class. The exercises will consist of individually tailored and targeted training for dynamic balance, muscle and bone strength, endurance, flexibility, gait and functional skills, as well as training to improve ‘righting’ or ‘correcting’ skills to avoid a fall, backward chaining (getting down to and up from the floor safely) and functional floor exercises. A full description of the original exercise programming and progression has been published.^34^

If required, participants will be provided with transport (taxi) to and from the classes and can bring along someone for support or bring a guide dog. Participants will be also be advised to exercise at home for 30 min to 2 hours a week (using a progressive adapted standardised home exercise programme that has been used in other related studies^31^ ^33^). Exercises will be provided in a large print booklet, DVD or audio format and will consist of specific chair-based exercises to improve strength and flexibility and standing exercises to improve balance, power and bone health, already practiced within the class setting.

### Data collection

Participants from the intervention and control groups will be telephoned each week by the study team for 24 weeks from baseline to record any adverse events, including falls that occur during the intervention period and for 12 weeks following the intervention. These will be assessed for duration, causality, expectedness, seriousness and severity as will any adverse events reported by the instructors within the exercise sessions. All non-serious adverse events (SAEs) will be reported on the study electronic case report form (eCRF) with hard copies sent to the Trial Management team within 4 weeks.

Table 1 offers a participant schedule of events.

**Table 1:** Violet study: participant schedule of events

			Baseline visit			Intervention or usual care/activities												Final follow-up
Venue	Prescreening	Initial screening	Site/home	Site		Site/home												Site/home
Time (weeks)	Post/hand-out	Telephone/site	0	0a	0b	1	2	3	4	5	6	7	8	9	10	11	12	24
Identification: invitation letters/PIS sent/handed out	X																	
Screening		X																
Informed consent			X															
Letter sent to GP				X														
Demographic data				X														
Comorbidities				X													X	X
Current medication				X													X	X
Socioeconomic information				X													X	X
FoF questionnaire				X													X	X
Timed Up & Go test				X													X	X
Outcome questionnaires				X													X	X
QoL questionnaires				X													X	X
Randomisation					X													
Falls diaries handed out				X					X				X				X	
Falls diaries collected									X				X				X	X
Structured interviews with PSIs					X												X	
Intervention: exercise sessions or usual activities						X	X	X	X	X	X	X	X	X	X	X	X	
Assessment of harms: phone calls						X	X	X	X	X	X	X	X	X	X	X	X	X
In-depth qualitative interviews with OPVI																	X	
Review of attendance records																	X	

FoF, fear of falling; GP, general practitioner; OPVI, older person visually impaired; PSI, postural stability instructor; QoL, quality of life.

### Data management and monitoring

Data will be entered on to a secure, password-protected, eCRF. As part of the data protection, confidentiality and anonymity requirements, all participants will be assigned a unique individual identifier. The database will be managed by the Clinical Trials Unit. Access will be limited to those deemed appropriate by the chief investigator. The research assistants, in conjunction with the database manager, and statisticians will carry out periodic data quality checks. These will include double data entry, snap shot analysis and query resolution. In relation to access to the final study data set, anonymised data will available from the chief investigator following an official request and ratification with the original members of the TOC, which was introduced in the ‘Study governance’ section above.

This is a low-risk trial and major safety data are not anticipated. The TOC will review any recorded SAEs (eg, injurious falls and/or hospitalisation due to falls) reported by the instructors within the exercise sessions or reported on the falls diaries returned by participants. The TOC will also monitor any trends in SAEs (eg, more than a few falls in the exercise groups during the exercise classes, or nearby, or a trend towards an increase in injury) and bring the intervention to a close, should this be deemed necessary. Overall the sponsor is responsible for auditing study conduct.

### Outcome measures

Standardised assessment instruments that have been used in falls research and with older people will be administered and are listed in box 1.^35–45^ The primary outcomes are related to feasibility, including the number of participants that are recruited (recruitment), that provide data at 24-week follow-up (retention), and attend a substantial amount (9/12) of the group exercise sessions if allocated to the intervention arm (adherence). Data will be collected at baseline, 12 and 24 weeks (box 1). The primary outcome of the future definitive RCT will be decided by the responsiveness to change, participant burden and participant feedback from this study.

Box 1 **Outcome assessment tools**

Fear of falling (Short Falls Efficacy Scale—International (FES-I)^35^);

Activity avoidance (2 questions^36^);

Balance/falls risk (Timed Up & Go test (TUG)^37^);

Falls Risk Assessment Tool (FRAT^38^);

Number of falls (falls diary and weekly telephone call^39^);

Self-reported physical activity (Phone-FITT^40^);

Loneliness (6-Item Scale for Overall Emotional and Social Loneliness^41^);

Anxiety and depression (Hospital Anxiety and Depression Scale (14 items)^42^);

Work and Social Adjustment Scale (WSAS^43^);

Quality of life (EQ-5D-5L^44^ and ICEpop CAPability measure for Older people (ICECAP-O)^45^);

Resource use via a health economic self-report service receipt inventory;

Adherence to the group exercise programme (register) and home exercise programme (self-reported).

FoF is likely to be selected as the primary outcome measure because while FoF is a common, debilitating and a significant predictor of a future fall particularly among older people,^46^ successful management of FoF is limited and unknown in VIOP. Recent reviews^26^ ^28^ ^30^ ^47^ show that there is a lack of high-quality research, which has FoF as a primary health outcome, provides guidance on routine practice in this area and translates research of FoF interventions to clinical settings. In addition, there is limited health economic data about FoF interventions.^28^ ^29^ Measuring the number of falls as a primary outcome does not take into account the more complex impacts of an intervention, which by reducing FoF, may increase participants' confidence in their ability to walk safely and continue to enjoy everyday activities. Assessing FoF before and after the proposed intervention captures participants' perceptions of change in their confidence to continue with physical and social activities. This in turn gives an indication of their perceived quality of life and whether the negative impacts of FoF, such as social isolation and risk of further falls, have been reduced.

## Qualitative evaluation of the feasibility study

### Interviews with VIOP

In-depth qualitative interviews will be conducted with a purposive sample of VIOP (∼3–5 from each site) to explore reasons for taking part; factors that facilitate/hinder them from participating in community-based falls prevention exercise groups; and experiences of recruitment and randomisation; views on outcome measures; experiences of the adapted intervention. The interviews with VIOP who complete the 12-week exercise programme (the intervention) will be conducted within 2 weeks following the end of trial data collection. Any VIOP who drops out during the intervention will also be contacted within 2 weeks of dropping out and asked if they would be willing to be interviewed by the researcher about their experiences of the intervention and the research and their reasons for dropping out.

The interviews will be conducted by the researchers in the form of a brief telephone conversation with a set of standardised questions, followed by, if willing, a face-to-face interview in a safe location chosen by the interviewee, such as their own home.

### Interviews with postural stability instructors

Structured interviews will be conducted with the PSIs. They will be interviewed at two points (before training and at the end of the intervention) to explore their (changing) perspectives on the provision of the intervention to VIOP over the duration of the intervention.

## Economic evaluation

A prospective economic evaluation will be rehearsed to develop and refine methods for a subsequent definitive trial. The main focus will be on how to accurately identify, quantify and value the additional costs of delivering the intervention and the potential resource implications versus usual ‘activity’ and on what measurement tools are appropriate to use with VIOP. The costing approach will incorporate a broad analytical perspective (NHS, social services and patient/carer costs), which will help to detect cost-shifting between sectors. Resources used in the exercise group will be identified in terms of capital equipment, staff time and travel/time for patients. Resource use in terms of out of pocket expenses will also be explored for all participants in addition to all treatment/care related to the intervention and any falls that may occur during the study period. This will be assessed retrospectively at the two follow-up periods (12 weeks and 6 months) by piloting the use of a falls resources/expenses form (to include informal caregivers time). This will facilitate the development of a reliable and valid tool to capture resource use. Appropriate unit costs to be applied to resource use will be identified. These will be sourced from a combination of local costings and national databases. Methods to value informal carer time will also be explored and defined, and all costs will be combined to rehearse the methods for total health, social care and patient/carer cost estimation in a subsequent definitive trial.

## Data analysis

As this is a feasibility study, a formal power calculation is not appropriate. We would aim to obtain a minimum of 30 responses in each trial arm at 6-month follow-up to estimate the critical parameters to the necessary degree of precision.^48^ To provide feasibility data, a total of 80 community-living VIOP aged 60+ will be recruited from NHS Low Vision Units and voluntary organisations to allow for loss to follow-up. As recruitment and retention rates are a relative unknown in this population, we will seek advice from the stakeholder groups on how best to reach them.

The main analyses will be descriptive, in order to inform the design of a future definitive study. We will calculate the numbers of eligible participants seen over the recruitment period, and the resulting rates of recruitment, compliance with randomisation, and data completion. We will also ascertain data completeness of the instruments and any potential bias in the completion of follow-up data to inform the choice of instruments in a future trial. The majority of the outcome data will be presented in simple descriptive tables presenting percentages, means and SDs or five-number summary (as appropriate), for each arm of the study. This information will be used to inform the design, choice of primary outcome, necessary sample size and approach to the analysis of the future definitive trial.

There is a potential for clustering effects, particularly class-based clustering in the intervention group and it will be part of the function of the pilot trial to investigate and estimate the size of any such effects. As this is a feasibility study, the results will provide estimates of variability in key outcomes both within and between classes and trial arms, which will be used to design a future definitive trial. However, there will be very limited information on the size of intraclass correlation coefficient (ICC) in a study with only two centres and two classes per centre, and any estimates of ICC based on this pilot trial will be very imprecise.

## Qualitative analysis

The interviews will be audio-taped and transcribed. Framework analysis will be used to analyse the interviews with the VIOP and PSIs.^49^ This has four non-linear interconnected stages: familiarisation of data, identifying a thematic framework, indexing data into the framework, developing charts from categories that are identified within the framework and finally mapping and interpretation. This will first be carried out within each site independent of the other and then combined with iterations until consensus is reached. The main themes and categories of the analysis will be shared with participants for member checking.

## Criteria for success

Following analysis of the qualitative interviews, the investigators will document the practicality of the study design, its impact on and acceptability to, both older people with visual impairment and professionals, and the acceptability and applicability of the outcome measures for older person visually impaired (OPVI). The conclusions from this discussion will inform the development of a definitive RCT and an intervention manual.

The progression criteria to judge the feasibility of progressing to a full trial will be based on:

1. ≥50% of OPVI eligible for the study willing to be recruited into the feasibility study;
2. ≥70% of the participants in the intervention arm complete 9/12 group sessions in the exercise programme (compliance);
3. Data collected on key outcomes at 6-month follow-up for ≥70% of those recruited;
4. <10% of SAEs deemed due to the intervention itself.

## Ethical approval

Glasgow Caledonian University was approved as a non-NHS site with local ethics approval. In addition, ethical approval was sought from the sponsoring institution: Northumbria University, and where needed the Research and Development department of the NHS Trust. As part of the ethical approval process, assurances were given regarding the processes in place to assure confidentiality and anonymity. Personal data will be regarded as strictly confidential. To preserve anonymity, any data leaving the site will identify participants using their unique study identification code only. The study will comply with the Data Protection Act, 1998. All study records and investigator site files will be kept at site in a locked filing cabinet with restricted access. No amendment to the protocol will be made without consideration and approval by the Trial Management Committee. Authorisation for any approval will be sought from the National Research Ethics Service (NRES) and NIHR. Reports regarding the progress of the study will be submitted as required to NRES and National Institute for Health Research (NIHR).

## Dissemination strategy

Results will be published in an open access peer-reviewed journal. The preliminary results from this feasibility study will be shared and discussed with the original stakeholder panel and the two charities involved. They will shape the dissemination strategy but this is likely to include articles and news items for vision charities and older people organisations, falls services, and disability and health policymakers.

## Discussion

The VIOLET study is designed to investigate the feasibility of a future definitive trial of the FaME exercise programme adapted for VIOP. There are potential risks and benefits to introducing exercises to this population. FoF in VIOP may exacerbate existing gait and balance difficulties, further increasing the risk of falls. Recent research has shown that older people are at increased risk of falling following intensive endurance exercise bouts,^50^ through exercise-induced alterations in respiration and muscular fatigue. It was noted that visual impairment could increase exercise-induced changes to postural control, and consequently the risk of falling.

This study will enable VIOP to collaborate with researchers and instructors to draw on their expertise and experience to adapt a commonly adopted exercise programme, that is known to reduce falls risk in high-risk frequent fallers^31^ and low-risk older adults,^33^ to their specific VI needs. This in turn may have positive impacts on gait and balance, increase confidence and lessen FoF. This study will add to an emerging body of work that is using FoF, as assessed by a widely validated cross-cultural tool (Falls Efficacy Scale International, FES-I), to address the gap in knowledge of how to manage FoF successfully.

The feasibility study will develop a manualised intervention, identify potential barriers and facilitating factors to recruitment and retention, and test aspects of the trial methodology to inform the design of a future definitive trial of an adapted exercise programme for VIOP.

## References

[1] Evans JR, Fletcher AE, Wormald RP Prevalence of visual impairment in people aged 75 years and older in Britain: results from the MRC trial of assessment and management of older people in the community. Br J Ophthalmol 2002;86:795–800. 10.1136/bjo.86.7.795 12084753 PMC1771210

[2] Ivers RQ, Cumming RG, Mitchell P Visual impairment and falls in older adults: the Blue Mountains Eye Study. J Am Geriatr Soc 1998;46:58–64. 10.1111/j.1532-5415.1998.tb01014.x 9434666

[3] Klein B, Moss S, Klein R Associations of visual function with physical outcomes and limitations 5 years later in an older population: the Beaver Dam eye study. Ophthalmology 2003;110:644–50. 10.1016/S0161-6420(02)01935-8 12689880

[4] Tinetti ME, Williams CS The effect of falls and fall injuries on functioning in community-dwelling older persons. J Gerontol A Biol Sci Med Sci 1998;53:M112–19. 10.1093/gerona/53A.2.M112 9520917

[5] Brouwer D, Sadlo G Limitations in mobility: experiences of visually impaired older people. Br J Occup Ther 2008;71:414–21. 10.1177/030802260807101003

[6] Salive ME, Gralnik J, Glynn RJ Association of visual impairment with mobility and physical function. J Am Geriatr Soc 1994;42:287–92. 10.1111/j.1532-5415.1994.tb01753.x 8120313

[7] Campbell S Deteriorating vision, falls and older people: the links. Visibility 2005 http://www.visibility.org.uk/what-we-do/research/#Falls (accessed 20 Oct 2015).

[8] Evans JR, Fletcher AE, Wormald RP Depression and anxiety in visually impaired older people. Opthalmology 2007;114:283–8. 10.1016/j.ophtha.2006.10.006 17270678

[9] Tinetti ME Clinical practice. Preventing falls in elderly persons. N Engl J Med 2003;348:42–9. 10.1056/NEJMcp020719 12510042

[10] Rubenstein LZ Falls in older people: epidemiology, risk factors and strategies for prevention. Age Ageing 2006;35(Suppl 2):ii37–41. 10.1093/ageing/afl084 16926202

[11] de Boer MR, Pluijm SM, Lips P Different aspects of visual impairment as risk factors for falls and fractures in older men and women. J Bone Miner Res 2004;19:1539–47. 10.1359/JBMR.040504 15312256

[12] Lamoureux E, Gadgil S, Pesudovs K The relationship between visual function, duration and main causes of vision loss and falls in older people with low vision. Graefes Arch Clin Exp Ophthalmol 2010;248:527–33. 10.1007/s00417-009-1260-x 20054556

[13] Lord SR, Smith ST, Menant JC Vision and falls in older people: risk factors and intervention strategies. Clin Geriatr Med 2010;26:569–81. 10.1016/j.cger.2010.06.002 20934611

[14] Steinman B, Nguyen A, Pynoos J Falls-prevention interventions for persons who are blind or visually impaired. Res Pract Vis Impairment Blindness 2011;4:83–91. PMC6309321 30595966

[15] Boyce T Falls—costs, numbers and links with visual impairment RNIB, Evidence and Service Impact. London: RNIB, 2011.

[16] Age UK. Falls in the over 65s cost £4.6 million a day. Age UK, 2015 http://www.ageuk.org.uk/latest-press/archive/falls-over-65s-cost-nhs (accessed 23 Nov 2015).

[17] Gillespie LD, Robertson MC, Gillespie WJ Interventions for preventing falls in older people living in the community. Cochrane Database Syst Rev 2009;(9):CD007146 10.1002/14651858.CD007146.pub2 19370674

[18] Gates S, Fisher JD, Cooke MW Multifactorial assessment and targeted intervention for preventing falls and injuries among older people in community and emergency care settings: systematic review and meta-analysis. BMJ 2008;336:130 10.1136/bmj.39412.525243.BE 18089892 PMC2206297

[19] Campbell AJ, Robertson MC, La Grow SJ Randomised controlled trial of prevention of falls in people aged ≥75 with severe visual impairment: the VIP trial. BMJ 2005;331:817 10.1136/bmj.38601.447731.55 16183652 PMC1246082

[20] Department of Health. The National Service Framework (NSF) for Older People NHS. London: Department of Health, 2001.

[21] NICE 21 Falls Guideline. Falls: assessment and prevention of falls in older people. National Institute of Clinical Evidence, 2004 Updated in 2010. http://guidance.nice.org.uk/CG21/Scope (accessed 20 Oct 2015).

[22] Lamb S, Gates S, Fisher J *Scoping exercise on fallers’ clinics, report* London: National Co-ordinating Centre for NHS Service Delivery and Organisation R & D, 2007.

[23] Brundle C, Waterman H, Ballinger C The causes of falls: views of older people with sight impairment. Health Expect 2015;18:2021–31. 10.1111/hex.12355 25736829 PMC4949546

[24] Yardley L, Smith H A prospective study of the relationship between feared consequences of falling and avoidance of activity in community-living older people. Gerontologist 2002;42:17–23. 10.1093/geront/42.1.17 11815695

[25] Grue EV, Finne-Soveri H, Stolee P Recent visual decline—a health hazard with consequences for social life: a study of home care clients in 12 countries. Curr Gerontol Geriatr Res 2010 10.1155/2010/503817 PMC2929488 20811648

[26] Zijlstra G, van Haastregt JC, van Rossum E Interventions to reduce fear of falling in community-living older people: a systematic review. J Am Geriatr Soc 2007;55:603–15. 10.1111/j.1532-5415.2007.01148.x 17397441

[27] Deandrea S, Lucenteforte E, Bravi F Risk factors for falls in community-dwelling older people: a systematic review and meta-analysis. Epidemiology 2010;21:658–68. 10.1097/EDE.0b013e3181e89905 20585256

[28] Parry S, Finch T, Deary V How should we manage fear of falling in older adults living in the community? BMJ 2013;346:f2933 10.1136/bmj.f2933 23714190

[29] Hadjistavropoulos T, Delbaere K, Fitzgerald TD Reconceptualizing the role of fear of falling and balance confidence in fall risk. J Aging Health 2011;23:3–23. 10.1177/0898264310378039 20852012

[30] Kendrick D, Kumar A, Carpenter H Exercise for reducing fear of falling in older people living in the community. Cochrane Database Syst Rev 2014;11:CD009848 10.1002/14651858.CD009848.pub2 PMC7388865 25432016

[31] Skelton D, Dinan S, Campbell M Tailored group exercise (Falls Management Exercise—FaME) reduces falls in community-dwelling older frequent fallers (an RCT). Age Ageing 2005;34:636–9. 10.1093/ageing/afi174 16267192

[32] Royal College of Physicians. Older people's experience of therapeutic exercise as part of a falls prevention service. London: R Coll P, 2012 http://www.laterlifetraining.co.uk/wp-content/uploads/2012/03/patient-and-public-involvement-report-march-2012.pdf (accessed 23 Nov 2015).

[33] Iliffe S, Kendrick D, Morris R Multicentre cluster randomised trial comparing a community group exercise programme and home-based exercise with usual care for people aged 65 years and over in primary care. Health Technol Assess 2014;18:vii–xxvii, 1–105 10.3310/hta18490 PMC4781144 25098959

[34] Skelton DA, Dinan SM Exercise for falls management: rationale for an exercise programme to reduce postural instability. Physiotherapy 1999;15:105–20. 10.1080/095939899307801

[35] Kempen GI, Yardley L, van Haastregt JC The Short FES-I: a shortened version of the falls efficacy scale-international to assess fear of falling. Age Ageing 2008;37:45–50. 10.1093/ageing/afm157 18032400

[36] Zijlstra GA, van Haastregt JC, van Eijk JT Prevalence and correlates of fear of falling, and associated avoidance of activity in the general population of community-living older people. Age Ageing 2007;36:304–9. 10.1093/ageing/afm021 17379605

[37] Podsiadlo D, Richardson S The timed ‘Up & Go’: a test of basic functional mobility for frail elderly persons. J Am Geriatr Soc 1991;39:142–8. 10.1111/j.1532-5415.1991.tb01616.x 1991946

[38] Nandy S, Parsons S, Cryer C, on behalf of the Falls Prevention Pilot Steering Group. Development and preliminary examination of the predictive validity of the Falls Risk Assessment Tool (FRAT) for use in primary care. J Public Health 2004;26:138–43. 10.1093/pubmed/fdh132 15284315

[39] Lamb SE, Jørstad-Stein EC, Hauer K, Prevention of Falls Network Europe and Outcomes Consensus Group. Development of a common outcome data set for fall injury prevention trials: the Prevention of Falls Network Europe consensus. J Am Geriatr Soc 2005;53:1618–22. 10.1111/j.1532-5415.2005.53455.x 16137297

[40] Gill DP, Jones GR, Zou GY The phone-FITT: a brief physical activity interview for older adults. J Aging Phys Act 2008;16:292–315. 18660552 10.1123/japa.16.3.292

[41] De Jong Gierveld J, Van Tilburg T A 6-item scale for overall, emotional, and social loneliness: confirmatory test on survey data. Res Ageing 2006;28:582–98. 10.1177/0164027506289723

[42] Zigmund AS, Snaith RP The hospital anxiety and depression scale. Acta Psychiatr Scand 1983;67:361–70. 10.1111/j.1600-0447.1983.tb09716.x 6880820

[43] Mundt JC, Marks LM, Shear MK The Work and Social Adjustment Scale: a simple measure of impairment in functioning. Br J Psychiatry 2002;180:461–4. 10.1192/bjp.180.5.461 11983645

[44] Herdman M, Gudex C, Lloyd A Development and preliminary testing of the new five-level version of EQ-5D (EQ-5D-5L). Qual Life Res 2011;20:1727–36 . 10.1007/s11136-011-9903-x 21479777 PMC3220807

[45] Davis JC, Liu-Ambrose T, Richardson CG A comparison of the ICECAP-O with EQ-5D in a falls prevention clinical setting: are they complements or substitutes? Qual Life Res 2013;22: 969–77. 10.1007/s11136-012-0225-4 22723152 PMC3672090

[46] Dionyssiotis Y Analyzing the problem of falls among older people. Int J Gen Med 2012;5:805–13. 10.2147/IJGM.S32651 23055770 PMC3468115

[47] Logghe IH, Verhagen AP, Rademaker AC The effects of Tai Chi on fall prevention, fear of falling and balance in older people: a meta-analysis. Prev Med 2010;51:222–7. 10.1016/j.ypmed.2010.06.003 20558197

[48] Lancaster G Design and analysis of pilot studies: recommendations for good practice. J Eval Clin Pract 2004;10:307–12. 10.1111/j..2002.384.doc.x 15189396

[49] Ritchie J, Lewis J, eds. Qualitative research practice. A guide for social science students and researchers. London: Sage Ltd, 2003.

[50] Donath L, Zahner L, Roth R Balance and gait performance after maximal and submaximal endurance exercise in seniors: is there a higher fall-risk? Eur J Appl Physiol 2013;113:661–9. 10.1007/s00421-012-2471-0 22915174

